# Retraction: microRNA-217 suppressed epithelial-to-mesenchymal transition through targeting PTPN14 in gastric cancer

**DOI:** 10.1042/BSR20193176_RET

**Published:** 2026-08-07

**Authors:** Gen Chen, Zhangshuo Yang, Maohui Feng, Zhiliang Wang

**Affiliations:** 1 Gui Zhou Provincial People's Hospital, Gui Yang, Gui Zhou, China; 2 Department of Gastroenterological Surgery, Zhongnan Hospital, Wuhan University, Wuhan, China; 3 Institute of Hepatobiliary Diseases, Transplant Center, Hubei Key Laboratory of Medical Technology on Transplantation, Zhongnan Hospital, Wuhan University, Wuhan, China

**Keywords:** EMT, gastric cancer, microRNA-217, PTPN14

This Retraction follows an Expression of Concern relating to this article previously published by Portland Press.

This article is being retracted from *Bioscience Reports* at the request of the Editor-in-Chief and the Editorial Board following the receipt of a notification from a reader, alerting the Editorial Board to duplications that occur between this article and other published articles. Upon investigation, additional duplications were identified. Specifically, these include:

- Overlaps between regions of the Figure 2A and 2B SGC-7901/MiR-217 control panels and the SC SGC-7901/Control panels from Figure 2B and 2C of Liu et al. 2017 (Eur Rev Med Pharmacol Sci 2017 Apr;21(8):1759-1767).
- Between the Figure 2A SGC-7901/MiR-217 Mimics panel and the Figure 2E Migration/miR-141 mimics panel from Zuo et al. 2015 (DOI: 10.1038/cddis.2014.573)
- Between Figure 3C GAPDH bands and the Figure 4A GAPDH bands (lanes 4 and 5) from Zhuang et al. 2014 (DOI: 10.1136/gutjnl-2014-307020)
- In Figure 5, third panel
  ◦ Between the N-cadherin bands and the Figure 4E p-Akt bands from Li et al. 2020 (DOI: 10.1016/j.advms.2020.03.005)
  ◦ Between the Vimentin bands and the Figure 4A ErbB4 bands (lanes 1-3) from Ryu et al. 2019 (DOI: 10.1371/journal.pone.0222587)
  ◦ Between the GAPDH bands and the Figure 4C IL10 bands (lanes 1-3) from Saha et al. 2019 (DOI: 10.1371/journal.pone.0218906)

The authors were contacted with regard to the concerns and provided raw data for the histology images, but concerns remain. As such, the Editorial Board stands by the decision to retract.

